# Electroanalytical investigation and voltammetric quantification of antiviral drug favipiravir in the pharmaceutical formulation and urine sample using a glassy carbon electrode in anionic surfactant media

**DOI:** 10.55730/1300-0527.3375

**Published:** 2022-02-23

**Authors:** Zeynep AKÇA, Hande İzem ÖZOK, Yavuz YARDIM, Zühre ŞENTÜRK

**Affiliations:** 1 Department of Analytical Chemistry, Faculty of Pharmacy, Van Yüzuncü Yıl University, Van, Turkey; 2 Department of Analytical Chemistry, Faculty of Science, Van Yüzüncü Yıl University, Van, Turkey

**Keywords:** Favipiravir, glassy carbon electrode, anionic surfactant, voltammetry

## Abstract

This work describes the electrochemical investigation of a promising antiviral agent, favipiravir (FAV) utilizing a nonmodified glassy carbon (GC) electrode, along with a unique voltammetric approach that can determine FAV with a good degree of accuracy, speed, and cost-effectiveness. Using cyclic voltammetry, the compound demonstrated a single well-defined and an irreversible oxidation peak at approximately +1.12 V (vs. Ag/AgCl) in *Britton*–*Robinson (BR)* buffer at pH 10.0. The synergistic effect of anionic surfactant, sodium dodecyl sulfate (SDS) on the adsorption ability of GC electrode remarkably increased the sensitivity of the stripping voltammetric measurements of FAV. Employing square-wave adsorptive stripping voltammetry at +1.17 V (vs. Ag/AgCl) (after 60 s accumulation at open-circuit condition) in BR buffer (pH 10.0) containing 3 × 10^−4^ M SDS, the linear relationship is found for FAV quantification in the concentration from 1.0 to 100.0 μg mL^−1^ (6.4 × 10^−6^–6.4 × 10^−4^ M) with a detection limit of 0.26 μg mL^−1^ (1.7 × 10^−6^ M). The proposed approach was used successfully to determine FAV in pharmaceutical formulations and model human urine samples.

## 1. Introduction

**Figure 1:**
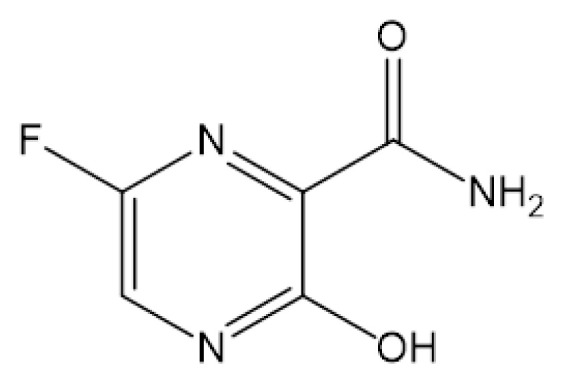
Chemical structure of favipiravir.

Favipiravir (FAV) is a purine nucleoside analogue that acts as a competitive inhibitor of RNA polymerase (Figure 1) [1]. FAV is unique among anti-flu drugs as its mechanism of action is through direct inhibition of viral transcription and replication. FAV is a new antiviral agent that selectively and potently inhibits the RNA-dependent RNA polymerase (RdRP) of influenza and various other RNA viruses. Because RdRP is absent in human cells and is conserved among RNA viruses, this different specific mechanism of targeting RNA viral polymerases makes FAV an attractive drug candidate. Therefore, a number of in vitro and in vivo works have been performed to demonstrate efficacy in cell culture and animal models [2].

SARS-CoV-2, a type of coronavirus that emerged in December 2019 in Wuhan, China, has been reported to have spread very rapidly around the world as of April 19, 2020. Intensive public health efforts are underway around the world to contain the COVID-19 outbreak. However, while treatments for COVID-19 continue to be defined, the use of existing antiviral agents against COVID-19 is being investigated. FAV is a promising broad-spectrum antiviral in the treatment of influenza virus infections, particularly because of the absence of drug-resistance mutations in cell culture or animal studies. As a result of in vitro studies, it has also been found to have an effect against influenza viruses resistant to oseltamivir and zanamivir, various viral hemorrhagic fever agents, SARS-CoV-2, including influenza A and B. In addition, FAV has been approved in Japan for new epidemic influenza strains that do not respond to standard antiviral therapies. Early treatment with FAV for COVID-19 is considered promising as a result of clinical studies [1,3].

In our literature review, various analytical methods have been reported for the quantification of FAV [4–10]. All of them were published within the last year (in 2021). Most of these studies use high performance liquid chromatography (HPLC) with different types of spectral detection. Although these commonly used techniques show high accuracy and sensitivity in analysis (especially coupled to tandem mass spectrometry), their applications often require expensive instruments, skilled personnel for operation, tedious time-consuming and extraction steps, long analysis times, and the use of environmentally unfriendly solvents.

When we survey the literature at the time of writing, it is seen that there are four voltammetric studies recommended for FAV determination in pharmaceutical and biological samples. At first, FAV was studied by our working group using electrochemically pretreated boron-doped diamond (BDD) electrode at pH 8.0 in the presence of cationic surfactant [11]. The second investigation was carried out on the screen-printed (SP) electrode modified with MnO_2_-rGO at pH 7.0 [12]. Another report was concerned with studying the determination of this compound at pH 4.0 using an electrochemical sensor based on the bimetallic nanocomposite modified glassy carbon (GC) electrode (Au@AgCSNPs/PEDOT: PSS/F-MWCNT/GC) [13]. Finally, by using carbon paste (CP) electrode modified with diamond nanoparticles (DNPs), FAV was detected in the tablet and serum samples [14].

Unlike many conventional methods, the sample can be analyzed directly without any pretreatment by using electroanalytical methods especially the voltammetric ones. Voltammetry can easily meet the needs in this field because this technique provides sufficient sensitive, satisfactory selective, precise, and reproducible results [15–19].

Development of new electrode material is very important to improve the performance of the voltammetric method. A simple and inexpensive way, apart from the electrode material, is to use surfactants. For electroanalytical purposes, surfactants are either mixed with the electrode material such as carbon paste or added at their submicelle concentrations to the electrode solution in the voltammetric cell [20]. Several research groups have studied the interaction of hydrophilic or hydrophobic substances with the different charged surfactants on the surface of bare or chemically modified carbon-based electrodes [21–29].

Considering the above information, in this study, the electrochemical properties of FAV will be investigated using GC electrode in the presence of anionic surfactant. The developed voltammetric method will be applied to pharmaceutical formulation and urine samples.

## 2. Experimental

### 2.1. Chemicals

The reference standard of FAV was provided from Atabay Pharmaceuticals and Fine Chemicals Inc. (Turkey), and utilized without having to be extra purified. The stock standard solution of 1.0 mg mL^−1^ FAV was made by dissolving in methanol and then storing in a volumetric flask at 4 °C to prevent degradation while not in use. Britton–Robinson (BR) buffer (0.04 M, pH 2.0–12.0) was prepared by using analytical-grade reagents (boric, acetic, and orthophosphoric acids) and purified water from the Millipore Milli-Q system (Millipore, resistivity ≥ 18.2 MΩ cm). To prepare the working and calibration solutions of FAV, its stock solution was diluted, just before use, with the supporting electrolyte at selected pH. The stock solutions (1 × 10^−2^ M) of anionic surfactant, sodium dodecylsulfate, SDS (90%, Merck), and cationic surfactant, cetyltrimethylammonium bromide, CTAB (99%, Sigma) were prepared in water and water-methanol mixture (90:10, v/v), respectively. All voltammetric measurements were performed three times under laboratory conditions.

### 2.2. Apparatus and measurements

Electrochemical experiments were performed using a μAutolab type III (Metrohm Autolab B.V., Utrecht, Netherlands) that was operated by the GPES software (Version 4.9). The Savicky and Golay algorithm was used to smooth all square wave (SW) voltammograms, and the moving average algorithm was used to adjust the baseline (peak width of 0.01 V). The traditional three-electrode configuration was adopted, using a GC working electrode (with the inner diameter of 3 mm, BAS MF 2012), an Ag/AgCl/3 M NaCl reference electrode (BAS, Model RE-1, USA) as well as the platinum counter electrode (BAS, MW-4130, USA). The pH values were measured using the pH meter model WTW InoLab720 equipped with the combined glass electrode. Before each measurement, the GC electrode was mechanically cleaned to obtain repeatable and stable results. For this purpose, the electrode surface was polished manually with aqueous slurry of alumina powder (diameter of 0.01 μm) on a damp smooth polishing cloth (BAS MF 1040). Its surface was then thoroughly rinsed with water and then cleaned in an ultrasonic bath to remove any residual alumina.

Initially, cyclic voltammetry (CV) was utilized to characterize the electrochemical behavior of FAV and determine the reaction kinetics on the GC electrode. Following this, square-wave adsorptive stripping voltammetry (SW-AdSV) was used to optimize the experimental parameters such as the supporting electrolyte at different pH values, the accumulation variables and the surfactant content to improve the analytical performance of the method for FAV determination. The practical application was assessed using SW-AdSV in SDS-containing solutions.

For the quantitative determination of FAV, three-electrode system was immersed into the voltammetric cell containing required aliquot of the FAV working solutions and BR buffer at pH 10 in the presence of 3 × 10^−4^ M SDS. After this step, during the preconcentration period (60 s), the selected preconcentration potential (open-circuit condition) was applied, while the solution was stirred at 500 rpm. Following a fixed rest period (10 s) to reach the equilibrium of the solution, anodic scanning was applied from 0.0 to +1.3 V utilizing the SWV approach. The optimized operating SW parameters for analysis of samples were as follows: frequency, 75 Hz, pulse amplitude, 40 mV, step potential, 10 mV.

### 2.3. Sample preparation

Favimol tablets (Neutec Co., Turkey) labeled as containing 200 mg of FAV were obtained from a local hospital in Van (Turkey). Ten tablets were precisely weighed and ground in a mortar. Its sufficient amount equivalent to 25 mg FAV was transferred into a 50-mL calibrated dark flask, which was then filled to the desired volume with methanol. The contents of the flask were stirred continuously for approximately 10 min in order to achieve total dissolution. A suitable volume of the sample solution (100 μL) was added to the voltammetric cell containing 10 mL of BR buffer at pH 10.0 in the presence of 3 × 10^−4^ M SDS. The contents of FAV in sample solutions were determined after its standard additions of 1.0, 2.5, 5.0, 7.5, 10.0, and 12.5 μg mL^−1^ (the final concentrations in the voltammetric cell).

In the following step, the applicability of the developed procedure to the human urine samples was also demonstrated. Drug-free samples were collected from a healthy a laboratory coworker (female, age 25 years) on the day of the experiment. One milliliter of FAV stock solution (1 mg mL^−1^) transferred to a test tube, and the volume was completed to 10 mL with the urine sample followed by vortexing for one min. The appropriate volume of the final mixture (250 μL) was transferred into the voltammetric cell containing 10 mL of same supporting electrolyte mentioned above. FAV-free sample of the same urine was used as a blank. All experiments were performed in triplicate, and the standard addition method was performed for the detection of FAV.

## 3. Results and discussion

### 3.1. Cyclic voltammetry on the GC electrode

**Figure 2:**
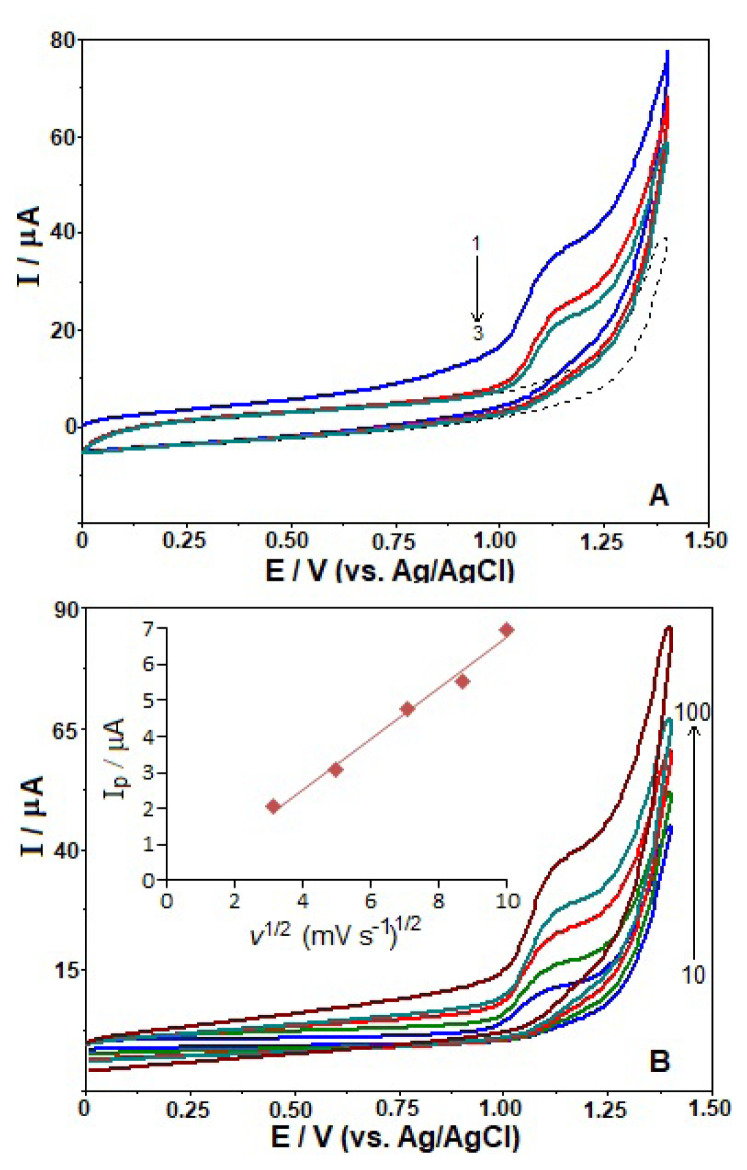
The repetitive CVs at the scan rate of 100 mV s^−1^ for 100 μg mL^−1^ favipiravir (A), and CVs at different scan rates (10, 25, 50, 75, and 100 mV s^−1^) for 100 μg mL^−1^ favipiravir (B) on the GC electrode in BR buffer at pH 10.0. A: Arrow indicates order of the recorded scans, dashed lines represent background current. B: Linear dependencies *i*_p_ vs. *v* ^1/2^ are appended in the inset.

The electrochemical behavior of FAV was first investigated by the CV technique without an accumulation step. Applying a scan rate of 100 mV s^−1^, three successive CV curves (CVs) for 100 μg mL^−1^ FAV were recorded in BR buffer (pH 10.0). As illustrated in Figure 2A, during the anodic sweep from 0.0 to +1.4 V, FAV exhibited one oxidation signal at about +1.12 V. On the other hand, the absence of a reduction peak in the reverse scan implies the irreversibility of electrode reaction of FAV. Passivation or contamination of the electrode surface may account for the decrease in its oxidation signals during the second and third potential cycles (more distinct in the case of second cycle).

To determine the GC electrode kinetics, the effect of the scan rates from 10 to 100 mV s^−1^ on the oxidation peak current response was examined for 100 μg mL^−1^ FAV in the above electrolyte solution (Figure 2B). As the potential scan rate increased, the peak intensities of the compound also increased. The anodic peak current (*i*_p_) of FAV was found to be proportional to the scan rate (*v*) using the equation *i*_p_ (μA) = 0.054 *v* (mV s^−1^) + 1.698, *r* = 0.993, which is suggestive of an adsorption-controlled electrode reaction of this compound. However, the plots of *i_p_* vs. *v*^1/2^ were also linear according to the following equation: *i*_p_ (μA) = 0.706 *v*^1/2^ (mV s^−1^) − 0.318, *r* = 0.995. It demonstrates a diffusion-controlled mechanism of the electrode reaction of FAV. To better understand the FAV oxidation on GC electrode, plots were also constructed between the log *i_p_* and log *v*. In this case, a linear relationship was achieved as follows: log *i*_p_ (μA) = 0.529 log *v* (mV s^−1^) − 0.231, *r* = 0.996.

In light of the above findings, we can hypothesize that the electrooxidation of FAV on the GC electrode can be regulated by both adsorption and diffusion, implying the presence of a mixing mechanism. Similar results have also been found in the previous works for some compounds using the various carbon-based electrodes [26,30,31].

**Figure 3:**
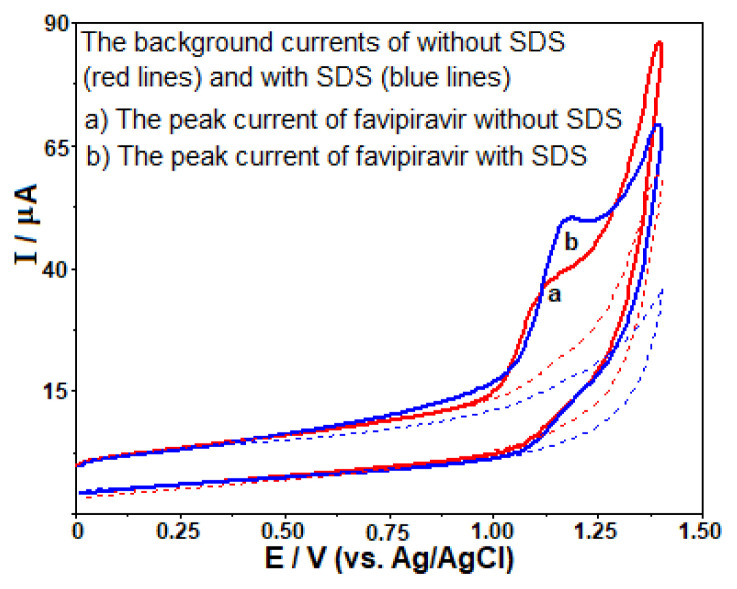
The CVs at the scan rate of 100 mV s^−1^ for 100 μg mL^−1^ favipiravir in the absence (a) and the presence of 3 × 10^−4^ M SDS (b) on the GC electrode in BR buffer at pH 10.0. The dashed lines represent background currents.

In order to examine the effect of anionic (negatively charged) surfactant SDS on the oxidation peak of FAV, it was added to the voltammetric cell containing BR buffer at pH 10.0, having a final concentration of 3 × 10^−4^ M (Figure 3). The CV curves showed that the peak current of the oxidation process of FAV increased being sharper when the solution contains SDS. More detailed investigations of the effect of this surfactant will be presented in the following sections.

### 3.2. Stripping voltammetry in the absence and the presence of surfactant

Taking into account the above experimental data, further work was dedicated towards studying the influence of pH of the BR buffer using SW-AdSV approach after carrying out an accumulation step on GC electrode. For this purpose, stripping measurements on 10 μg mL^−1^ FAV were carried out in the potential ranges from 0.0 to +1.6 V (in acidic region) and from 0.0 V to +1.3 V (in alkaline region), with an open-circuit accumulation at 30 s.

**Figure 4:**
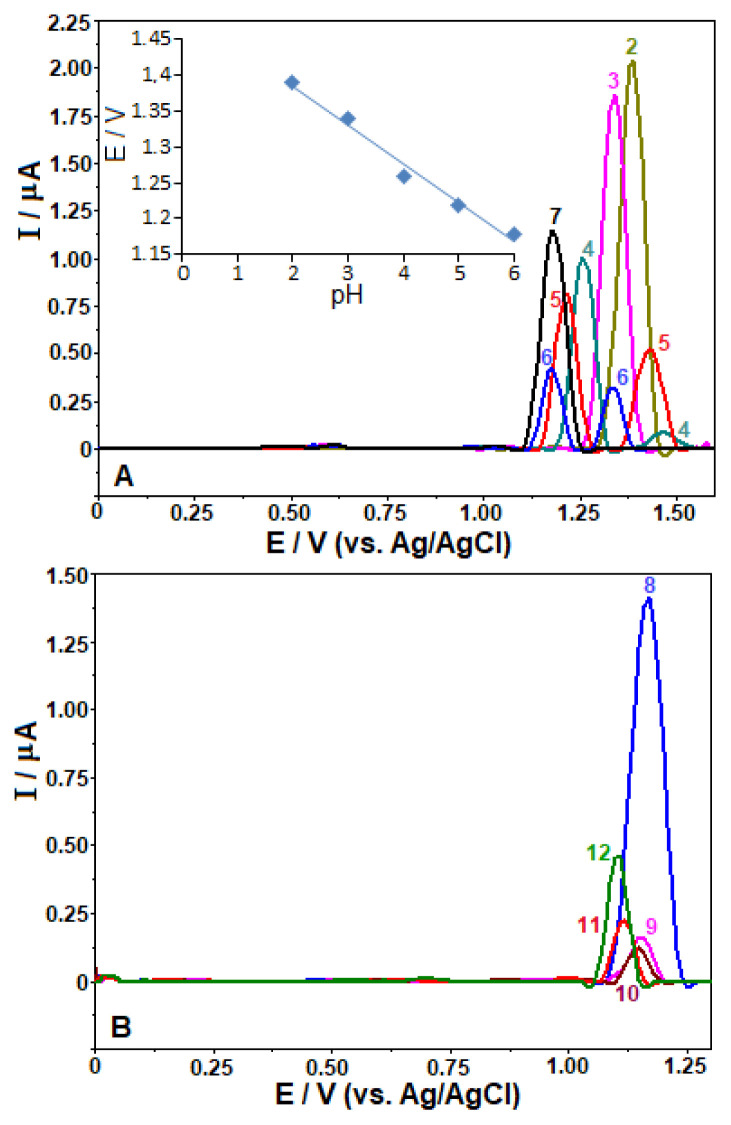
SW stripping voltammograms for 10 μg mL^−1^ favipiravir in BR buffer pH 2.0–7.0 (A) and pH 8.0–12.0 (B) on the GC electrode. Accumulation period 30 s at open-circuit condition; SWV parameters: frequency, 75 Hz; step potential, 8 mV; pulse amplitude, 30 mV. A: Inset depicts the plot of E_p_ vs. pH.

As can be seen from Figure 4A, FAV gave rise a single anodic peak for a solution with pH 2.0 and 3.0. Meanwhile, two peaks were recorded between pH 4.0 and 6.0, together with a significant reduction of their peak currents. At pH 4.0, the height of the peak observed at less positive potential was remarkably higher than that of other one. As pH increased, its intensity decreased and the second one increased. When the experiments were performed in neutral and alkaline solutions over the pH range 7.0–12.0 (Figures 4A and 4B), these two peaks were fused into again a single peak at less positive potentials that is accompanied by a maximum increase in the anodic peak currents with increasing pH up to 8.0, and then decreased significantly with different degrees at higher pH values. It is noteworthy that such splitting was not observed in our previous study carried out BDD electrode [11], which is attributed to the importance of different allotropes of carbon electrode material involving in the electrode reaction.

On the other hand, increasing the solution pH from 2.0 to 6.0 promoted displacements in the peak potential (*E*_p_) to less positive values, which can be expressed by the following equations: *E*_p_ (V) = −0.054 pH + 1.495 (R^2^ = 0.982). This behavior reveals the proton-dependent nature of FAV on the GC electrode. The slope of 54 mV/pH is close to the theoretical value of 59 mV, suggesting that the electrode reaction involves the equal numbers of electrons and protons. Above pH 7.0, the potential of the oxidation peak becomes nearly pH-independent (from +1.18 to +1.10 V), indicating that no proton transfer step occurs before the electron transfer rate-determination step at these pH values.

At this point, it should be underlined that FAV belongs to the potentially tautomeric pyrazinecarboxamide family. Considering the knowledge from our recently published paper on the determination of FAV [11], the break observed in the *E*_p_/pH plot at about pH 7.0, which is consistent with the clear change in the peak intensity can be explained by the replacement of the ketone form with the enol form (by intramolecular transferring of a proton) in the tautomeric equilibrium of FAV.

On the other hand, to clarify the exact electrooxidation mechanism of FAV, more experimental research should be carried out using a galvanostatic/potentiostatic coulometry with subsequent spectral analysis. However, remembering the reductive behavior of nonoxidized pyrazinamide [32,33], and considering that this compound differs from FAV in the absence of the hydroxyl group in the pyrazine ring, it may be assumed that the introduction of hydroxyl group plays an important role in the oxidation process of FAV molecule.

**Figure 5:**
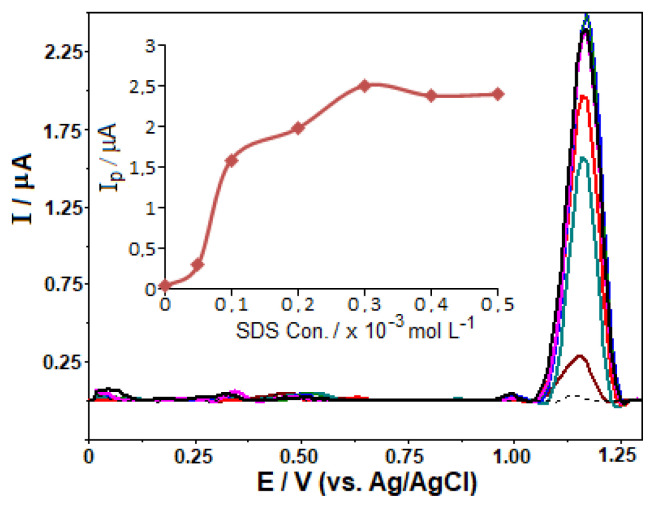
SW stripping voltammograms for 10 μg mL^−1^ favipiravir in BR buffer pH 6.0–12.0 in the presence of 3 × 10^−4^ M SDS on the GC electrode. The other operating conditions as indicated in Figure 4.

As soon as the *i*_p_/pH relation was studied, the attention was then turned to the effect of anionic surfactant (SDS) in order to develop a sensitive analytical methodology. For this purpose, fixing FAV concentration at 10.0 μg mL^−1^, SDS were added to the BR buffer solution with different pH values from 2.0 to 12.0, having its final concentration of 3 × 10^−4^ M (Figure 5). Voltammograms recorded in surfactant-containing solutions with pH values from 2.0 to 5.0 showed higher background current which prevented the correct measurement of the peak current height. At pH ≥ 6.0, an increase in peak current of varying degrees was observed with the addition of SDS, surprisingly reaching the highest value at pH 10 (3.36 μA at +1.17 V).

**Figure 6:**
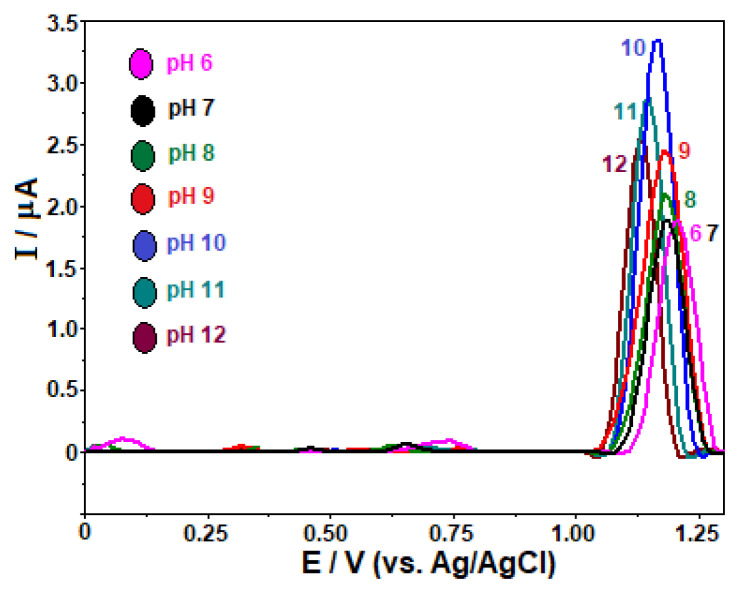
SW stripping voltammograms for 5 μg mL^−1^ favipiravir in BR buffer at pH 10.0 in the presence of various SDS concentrations (5.0 × 10^−5^–5 × 10^−4^ M) on the GC electrode. The voltammogram without SDS is represented using the dashed lines. Inset: plot of *i*_p_ against the concentration of SDS. The other operating conditions as indicated in Figure 4.

After this preliminary study, in the next step, keeping the FAV concentration constant at 5.0 μg mL^−1^ in BR buffer at pH 10.0, the concentration of SDS was increased from 5.0 × 10^−5^ to 5 × 10^−4^ M. As can be seen in Figure 6, an important signal enhancement in peak current was observed in the cooperation of SDS. The intensities increased gradually with SDS concentration up to 3.0 × 10^−4^ M. At its higher concentrations, it was not observed any significant change in the signal (Figure 6 inset). Regarding the peak potentials, its values remained practically constant when the electrolyte solution contains SDS.

Furthermore, the effect of cationic surfactant (cetyltrimethylammonium bromide, CTAB) was also explored on the voltammetric response of FAV at pH 10.0. After its addition to the electrolyte solution at its concentration of 3.0 × 10^−4^ M, the peak currents were found nearly the same as that obtained in its absence, with a slight shift in the peak potential to more positive value.

To our knowledge, a detailed scientific work for the determination of p*K*_a_ values of FAV has not been documented so far. This may be explained by the complexity of the proton transfer process, which may involve several complex steps to form various keto and enol tautomers (mentioned above) of FAV. Considering this uncertainty in the literature, the interaction mechanism of FAV with anionic surfactant SDS adsorbed on the GC electrode (electrostatic/hydrophobic interactions or coadsorption/adsolubilization), and thus the reason for the significant increase in its peak current at pH 10.0 remain unclear.

To sum up, all experiments were performed by fixing the concentration of SDS at 3.0 × 10^−4^ M for the rest of present analytical investigation. It should be highlighted that by applying the preconcentration/stripping process after SDS addition, FAV signals were almost 10 times higher compared to the measurements in its absence.

Taking into consideration the adsorptive features of FAV on the GC electrode, further work was dedicated toward studying the effect of preconcentration/stripping conditions, such as accumulation time (*t*_acc_) as well as accumulation potential (*E*_acc_) (data not shown) for 5.0 μg mL^−1^ FAV under the optimum experimental conditions. The effect of the *t*_acc_ upon the oxidation signal was investigated in the range 0–240 s at open-circuit state. The results showed that the oxidation peak currents reach a maximum value at 60 s of accumulation, beyond which it remained constant, indicating that electrode surface becomes saturated with the FAV molecules. On the other hand, the dependence of the anodic peak current on the *E*_acc_ was examined either at open-circuit condition or over the potential range from +0.1 to +1.0 V with a *t*_acc_ of 60 s. In this case, the anodic peak current remained almost unchanged in the whole range, revealing that this parameter had little effect on the determination of FAV, thus open-circuit accumulation was selected.

The dependence of stripping responses on SWV operating parameters such as frequency (*f* = 25–125 Hz), step potential (Δ*E*_s_ = 6–14 mV) and pulse amplitude (Δ*E*_sw_ = 30–70 mV) was finally demonstrated under the above conditions (data not presented) in order to optimize the experimental set-up for FAV determination. The optimization was carried out in such way that one parameter is always changed while other two parameters are kept constant. For entire analysis, the optimized values were: *f*, 75 Hz; Δ*E*_s_, 10 mV; and Δ*E*_sw_, 40 mV.

### 3.3. Quantification of favipiravir using GC electrode in the presence of SDS

**Figure 7:**
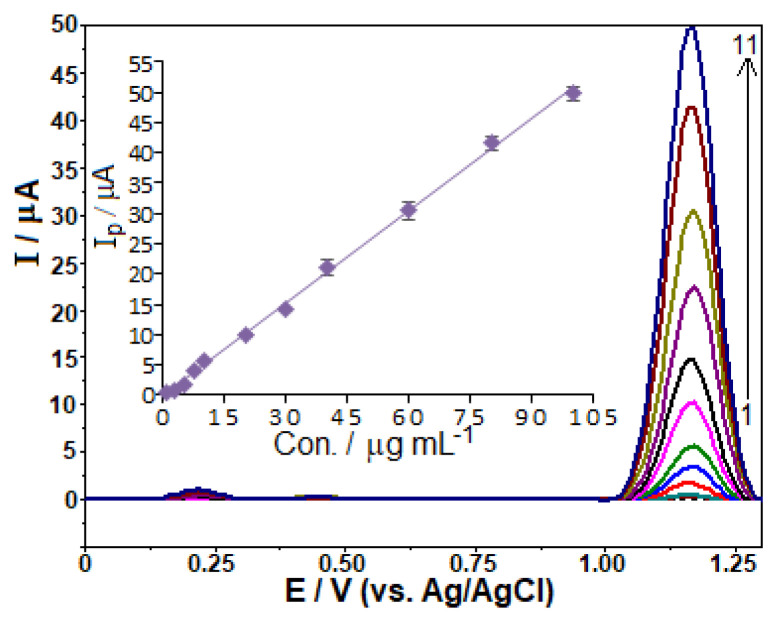
SW stripping voltammograms for favipiravir levels of (1–11) 1.0, 2.5, 5.0, 7.5, 10.0, 20.0, 30.0, 40.0, 60.0, 80.0, and 100.0 μg mL^−1^ in BR buffer at pH 10.0 containing 3 × 10^−4^ M SDS. Accumulation time 60 s at open-circuit condition; SWV parameters: frequency, 75 Hz; step potential, 10 mV; pulse amplitude, 40 mV. Inset shows the corresponding calibration plot for the quantitation of favipiravir.

To assess the analytical performance, the oxidation peak current was examined in relation to FAV concentrations using the previously optimized experimental and operating conditions. Figure 7 presents the respective stripping voltammograms and corresponding calibration curve. It is seen from the figure that the calibration graph consists of a linear segment, from 1.0 to 100.0 μg mL^−1^ (6.4 × 10^−6^−6.4 × 10^−4^ M) with its good linearity according to the following equation:

*i*_p_ (μA) = 0.509 C (μg mL^−1^) − 0.189 (*r* = 0.999, *n* = 11), where *i*_p_ is the anodic peak current, *r* the correlation coefficient and *n* the number of experiments.

The limits of detection (LOD) and quantification (LOQ) were found to be 0.26 μg mL^−1^ (1.7 × 10^−6^ M) and 0.87 μg mL^−1^ (5.5 × 10^−6^ M), respectively. The LOD and LOQ were estimated with the formula 3 *s*/*m* and 10 *s*/*m*, respectively, which takes into account the standard deviation of the ten measurements of the lowest concentration within the calibration range, as well as the slope of the calibration curve.

The comparison between the analytical performance of the previously reported works and the study here reported for the voltammetric quantification of FAV is given in Table 1. From these data, it can be clearly seen that the developed voltammetric method exhibits lower sensitivity (in terms of LOD) for the detection of FAV compared to other four techniques. However, it is worth noting at this point that these literature techniques use modified carbonaceous electrodes except that reported in [11]. However, the modification process requires high cost modifiers and prolonged preparation, often leading to poorly reproducible results.

To determine the precision of the developed approach, the intraday (ten replicates) as well as interday (three days) reproducibility of 1 μg mL^−1^ FAV was examined under identical conditions. The relative standard deviation (RSD) values were found as 4.41% and 6.26%, respectively. These data suggest that the GC electrode is a suitable electrode material for the repeatable FAV measurements in real samples.

Before proceeding to the analysis of the samples, the influence of possibly interfering substances, most of which were found in tablet formulations or biological samples was determined for 10 μg mL^−1^ (approximately 6.4 × 10^−5^ M) FAV under the above experimental settings. Using the indicated interfering chemicals, the tolerance limit was set to a concentration that produced an average error of ±7% in the oxidation peak current of FAV. The obtained results were evaluated by using calculated the oxidation current changes of FAV, and given in Table 2. It was shown that inorganic ions such as Na^+^, K^+^, Mg^2+^, Ca^2+^, Mn^2+^, Cu^2+^, Fe^3+^, Al^3+^, Ti^4+^, Cl^-^, NO_3_^−^and SO_4_^2−^ did not have a significant effect on the oxidation peak current when their 100-fold excess amounts were used. It was observed that sugars such as lactose, glucose and fructose had no effect on FAV oxidation peaks even when 100 times more was added to the mixture. Furthermore, the effects of compounds included in pharmaceutical formulations such as microcrystalline cellulose, corn starch and magnesium stearate on the oxidation current responses of FAV were negligible. The effect of dopamine, ascorbic acid, and uric acid, which could be present in biological fluids, were tested for their molar concentrations at the ratios of 1:1, 1:10, and 1:25 (FAV: interfering agent). The obtained results showed that there were no significant effects from these compounds on the oxidation curve of FAV. Since paracetamol is widely used as an analgesic and antipyretic drug, its effect on FAV oxidation signal was also investigated, and no effect was observed at its 25-fold excess.

In the light of the above results, the final step is the examination of the practical applicability of proposed approach in commercially available pharmaceutical formulations and spiked human urine samples. The preparations of the samples are discussed in the Experimental Section.

**Figure 8:**
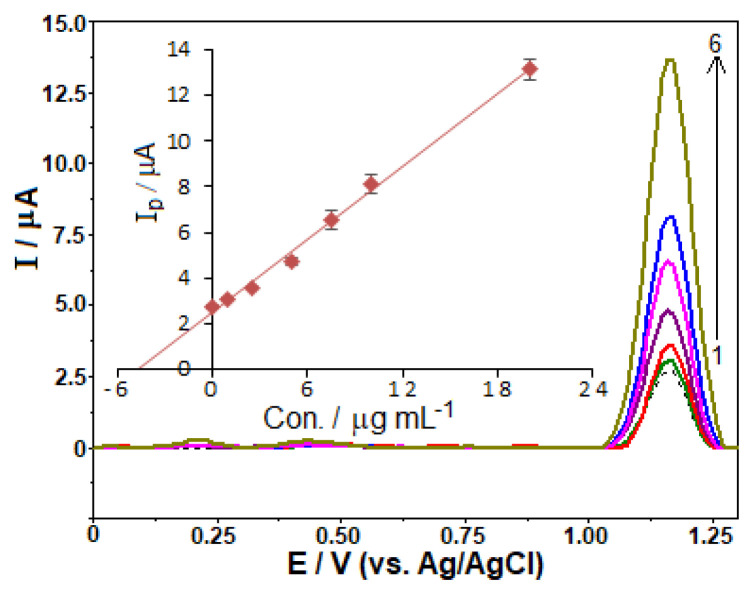
SW stripping voltammograms for diluted Favimol tablet sample (dashed lines) and after standard additions of 1.0, 2.5, 5.0, 7.5, 10.0, and 12.5 μg mL^−1^ (1–6) favipiravir in BR buffer at pH 10.0 containing 3 × 10^−4^ M SDS on the GC electrode. The other operating conditions as indicated in Figure 7.

Tablet samples were analyzed without any sample extraction, required in chromatographic techniques. Figure 8 depicts the representative stripping voltammograms for the analysis of tablet samples. Using the related graphical evaluation of multiple standard addition method [*i*_p_ (μA) = 0.531 C (μg mL^−1^) + 2.489 (*r* = 0.999)], and taking into account adequate dilutions, FAV content was found to be 187.6 mg per tablet (RSD of 3.3%), which approaches the label value of 200.0 mg declared by the provider.

In order to verify the validity of the newly developed method for practical applications, spike/recovery experiments were carried out by adding the standard FAV solutions (final concentrations of 1, 5, and 10 μg mL^−1^) to 10 mL of tablet sample solution in voltammetric cell, and voltammetric responses were measured. As shown in Table 3, no significant matrix influence was observed on the proposed voltammetric technique.

**Figure 9:**
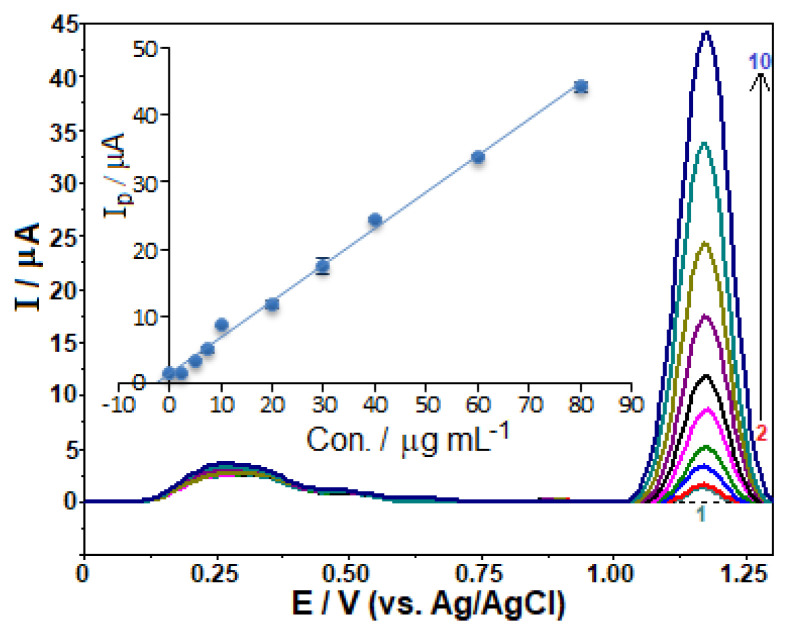
SW stripping voltammograms of model human urine sample from volunteer (diluted with supporting electrolyte in the ratio of 1:40, v/v): dashed line depicts only urine sample without favipiravir, number 1 shows in the presence of 2.5 μg mL^−1^ favipiravir in the urine sample, numbers from 2 to 9 shows after standard additions of 2.5, 5.0, 7.5, 10.0, 20.0, 30.0, 40.0, 60.0, and 80.0 μg mL^−1^ favipiravir. Inset depicts the result of analysis by standard addition method. Other operating conditions as indicated in Figure 7.

The capacity of the proposed method was also tested for the analysis of more complex matrices such as spiked human urine samples without any complex treatment or separation. Figure 9 shows the stripping curves (with a spiking FAV concentration of 2.5 μg mL^−1^ in the electrochemical cell) and graphical evaluation of multiple standard addition method. In urine sample before spiking, there was a detectable peak at around +0.25 V (seen more clearly in original curves), which could be due to the oxidation of uric acid. After spiking, the distinct peak observed at +1.17 V could be related to the oxidation of FAV. The peak intensity of the electrochemical process increased proportionally after eight additions of standard FAV solution at final concentrations ranging from 2.5 to 80 μg mL^−1^ (the inset of Figure 9), yielding a linear calibration plot; *i*_p_ (μA) = 0.538 C (μg mL^−1^) + 1.281 (*r* = 0.999). As shown in Table 4, the developed approach offers a satisfactory recovery and RSD value, demonstrating its potential applicability for the analysis of such a complex matrix.

At this point, it is noteworthy to underline that the administered dose of FAV is eliminated significantly in urine as unchanged drug [34]. However, the FAV concentration level in urine samples from patients infected with influenza is expected to be lower than the LOD value of the method developed in this study. Bearing in mind that there is no chromatographic technique coupled with amperometric detection, in this case, sensitivity can be achieved using the separation power of the HPLC with the combination of electrochemical detection on the GC electrode.

## 4. Conclusion

Considering very few reports dealing with the determination of a novel antiviral prodrug FAV, in the present study, the capability of the modification-free GC electrode in SDS-containing media was introduced for its electrochemical investigation and voltammetric quantification in pharmaceutical and biological samples.

## Figures and Tables

**Table 1:** Comparison of the analytical performance of the proposed approach with previously reported voltammetric methods for the determination of favipiravir.

Electrode	Linearity range (M)	LOD (M)	Ref.
BDD (in the presence of CTAB)	6.4 × 10^−8^–6.4 × 10^−7^	1.8 × 10^−8^	[11]
MnO_2_-rGO/SP	1.0 × 10^−8^–5.5 × 10^−5^	9.0 × 10^−9^	[12]
Bimetallic nanocomposite/GC	5.0 × 10^−9^–9.0 × 10^−9^ 9.0 × 10^−9^–1.9 × 10^−6^	4.6 × 10^−10^	[13]
NPs/CP	2.0 × 10^−7^–1.0 × 10^−6^ 8.0 × 10^−7^–6.0 × 10^−6^	4.8 × 10^−9^ 2.4 × 10^−7^	[14]
GC (in the presence of SDS)	6.4 × 10^−6^–6.4 × 10^−4^	1.7 × 10^−6^	This work

BDD, boron doped diamond; CTAB, cetyltrimethylammonium bromide; MnO_2_-rGO/SP, MnO_2_-reduced graphene oxide modified screen-printed; NPs/CP, diamond nanoparticles modified carbon paste; GC, glassy carbon; SDS, sodium dodecylsulfate.

**Table 2:** The influence of potential interfering agents on the current response of favipiravir.

Interference	Concentration ratios (FAV: interference)	Current change (%)
The inorganic ions	1:100	< 6
The sugars	1:100	< 6
Microcrystalline cellulose	1:50	< 3
Corn starch	1:50	< 3
Magnesium stearate	1:50	< 4
Ascorbic acid	1:25	< 3
Uric acid	1:25	< 4
Dopamine	1:25	< 4
Paracetamol	1:25	< 3

Inorganic ions; Na^+^, K^+^, Mg^2+^, Ca^2+^, Mn^2+^, Cu^2+^, Fe^3+^, Al^3+^, Ti^4+^, NO_3_^−^, SO_4_^2−^, Cl^−^, Sugars; lactose, glucose, fructose.

**Table 3:** Analysis of tablet samples spiked with favipiravir standard solutions using proposed voltammetric method .

Added^a^ (μg mL^−1^)	Expected^a^ (μg mL^−1^)	Found^a^,^b^ (μg mL^−1^)	Recovery (%) ± RSD (%)
0	–	4.69	0 ± 2.58
1.0	5.69	6.06	106.5 ± 4.12
5.0	9.69	9.82	101.3 ± 3.61
10.0	14.69	14.34	97.6 ± 3.21

a Concentration in the measured solution.

b Average of three replicate measurements.

**Table 4:** Measurement results for addition and recovery of favipiravir from human urine sample using the proposed voltammetric method.

Added (μg mL^−1^)	Found^a^ (μg mL^−1^)	Recovery (%) ± RSD (%)
2.50	2.38	95.2 ± 3.6

a Calculated by the use of standard addition method.

Values reported are the average of three independent analyses of the same sample.
